# On the feasibility of wireless radio frequency ablation using nanowire antennas

**DOI:** 10.1063/5.0053189

**Published:** 2021-07-01

**Authors:** Nicholas J. Rommelfanger, Guosong Hong

**Affiliations:** 1 Department of Applied Physics, Stanford University, Stanford, California 94305, USA; 2 Wu Tsai Neurosciences Institute, Stanford University, Stanford, California 94305, USA; 3 Department of Materials Science and Engineering, Stanford University, Stanford, California 94305, USA

## Abstract

Radio frequency ablation (RFA) is a proven technique for eliminating cancerous or dysfunctional tissues in the body. However, the delivery of RFA electrodes to deep tissues causes damage to overlying healthy tissues, while a minimally invasive RFA technique would limit damage to targeted tissues alone. In this manuscript, we propose a wireless RFA technique relying on the absorption of radio frequencies (RFs) by gold nanowires *in vivo* and the deep penetration of RF into biological tissues. Upon optimizing the dimensions of the gold nanowires and the frequency of the applied RF for breast cancer and myocardium tissues, we find that heating rates in excess of 2000 K/s can be achieved with high spatial resolution *in vivo*, enabling short heating durations for ablation and minimizing heat diffusion to surrounding tissues. The results suggest that gold nanowires can act as “radiothermal” agents to concentrate heating within targeted tissues, negating the need to implant bulky electrodes for tissue ablation.

## I. INTRODUCTION

Radio frequency ablation (RFA) is a minimally invasive procedure in which an alternating current with a frequency of 400–500 kHz is passed through an electrode to produce Joule heating and destroy dysfunctional tissues *in vivo*.^1^ Depending on the type of dysfunctional tissue targets, RFA has been performed to destroy abnormal electrical pathways in the cardiac tissue,^2–4^ cancerous tissues,^5,6^ and renal sympathetic nerves contributing to refractory hypertension.^7,8^ One of the critical challenges of RFA implantation arises from the use of large electrodes with a tip size of a few millimeters, thus resulting in excessive tissue damage during insertion and removal as well as limited spatial resolution of the ablation.^9^ Furthermore, the insertion of RFA electrodes requires an invasive procedure to dissect the superficial tissues since the alternating current needs to be delivered to the tip of the electrode via a wired interface.^10^

To address these challenges, the Rogers group has incorporated microelectrodes for RFA into multifunctional balloon catheters, leveraging the minimally invasive delivery of catheter tubing via the circulatory system.^2,3^ Specifically, a two-dimensional (2D) array of 8 × 8 stimulation electrodes is incorporated into a soft electronics platform, which facilitates the conformal mounting of these electrodes onto the curvilinear surface of a balloon catheter.^3^ Spatially programmable RFA can be applied by delivering currents to selected electrodes in the array, with temperatures mapped simultaneously by a built-in array of temperature sensors in the same multifunctional catheter. The high spatial resolution of RFA is enabled by the 64 microelectrodes in the multifunctional balloon catheter, thus providing a promising alternative to clinical RFA practice in which lesions are created by manually moving a single-channel RFA electrode.

Motivated by the reduced invasiveness and the improved spatial resolution of catheter-incorporated RFA electrodes, we ask if RFA therapy can be delivered via a wireless interface, capitalizing on the free-space transmission and deep-tissue penetration of electromagnetic waves in the radio frequencies (RFs).^11^ Free-space RF radiation has been used in the clinic for hyperthermia cancer treatment, but the achievable spatial resolution is diffraction-limited by the long wavelengths of RF. We have recently demonstrated that prolate metal spheroids with high aspect ratios, approximating nanowires, strongly absorb RF radiation and produce significant differential heating over biological tissues in an RF field.^12^ Building on this recent finding, we hypothesize that these prolate metal spheroids can act as nanoantennas to concentrate the incident free-space RF radiation, thus producing effective RFA with high spatial resolution. Moreover, the demonstrated aqueous suspendability of these metal nanowires enables minimally invasive syringe injection of these nanoantennas into specific tissue targets via a hypodermic needle.^13^ Therefore, metal nanowires provide an attractive alternative to the conventional means of implementing RFA for region-specific ablation of dysfunctional tissues *in vivo*.

**FIG. 1.**
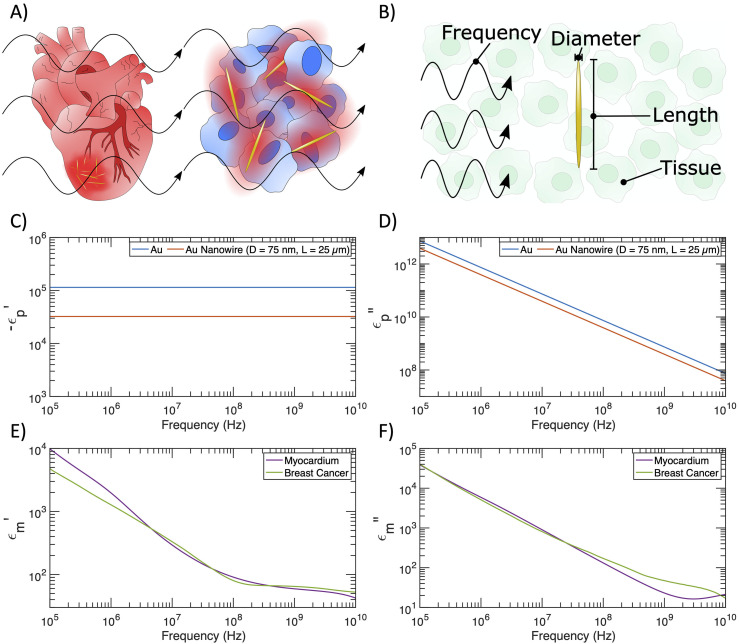
Dielectric properties of gold nanowires, the myocardium, and the breast cancer tissue. (a) Schematic of differential heating caused by RF irradiation of metallic prolate spheroids on the surface of the heart and in a tumor. (b) Tunable variables for relative absorption ratio calculations with gold prolate spheroids. (c) Negative real component of the dielectric function vs frequency for bulk gold and a gold nanowire with diameter D= 75 nm and length L= 25 *μ*m. (d) Imaginary component of the dielectric function vs frequency for bulk gold and a gold nanowire with diameter D= 75 nm and length L= 25 *μ*m. (e) Real component of the dielectric function vs frequency for myocardium and breast cancer tissues. (f) Imaginary component of the dielectric function vs frequency for myocardium and breast cancer tissues.

In this paper, we develop a theoretical framework to elucidate the feasibility of wireless RFA *in vivo* via free-space RF radiation. Specifically, we provide numerical simulations of gold nanowires with achievable dimensions in breast cancer and myocardial tissues under several frequencies common in hyperthermia treatments and RFA: 13.56 MHz, 434 MHz, and 400 kHz [Fig. 1(a)]. We use an analytical equation derived in our recent work^12^ to calculate the differential heating of gold nanowires in these two types of tissues, followed by solving the Pennes bioheat equation to compute the spatial distribution of temperature increases as a function of time under RF irradiation. Our results show that a tissue heating rate much higher than that of conventional RFA approaches can be achieved with comparable spatial resolution by free-space RF irradiation in the presence of gold nanowire nanoantennas. Our study provides a practical guide for choosing the appropriate sizes of gold nanowires to afford effective tissue ablation *in vivo* under RF irradiation of various frequencies.

## II. RESULTS

### A. The dielectric properties of gold nanowires and biological tissues of interest

Prolate metal spheroids of appropriate dimensions act as nanoantennas to concentrate impinging RF energy, thus producing local heating. The ability of these nanoantennas to concentrate RF energy relative to RF absorption by endogenous biological tissue is quantified as the relative absorption ratio $F_{abs}$. $F_{abs}$ is defined as the ratio of $\left\langle C_{abs}\right\rangle$, the absorption cross section of a nanoantenna (averaged over all possible particle orientations), to $C_{amb}$, the absorption cross section of the (ambient) endogenous tissue with the same volume.^12,14^ For a volume of arbitrary material, the absorption cross section itself is calculated by normalizing the power loss within the volume to the irradiance of the incident wave.^15^ In our recent work, the analytical expression of $F_{abs}$ is shown as follows:^12^

$$F_{abs}=\frac{\left\langle C_{abs}\right\rangle }{C_{amb}}=\frac{1}{3}\sum_{i=1}^{3}\frac{1}{{L_{i}}^{2}}\frac{{\left|\epsilon_{m}\right|}^{2}}{{\epsilon_{m}}^{\prime \prime }}\frac{{\epsilon_{p}}^{\prime \prime }}{{\left|\epsilon_{p}-\epsilon_{m}\left(\frac{L_{i}-1}{L_{i}}\right)\right|}^{2}},$$ (1)

where $\epsilon_{p}={\epsilon_{p}}^{\prime }+i{\epsilon_{p}}^{\prime \prime }$ and $\epsilon_{m}={\epsilon_{m}}^{\prime }+i{\epsilon_{m}}^{\prime \prime }$ denote the complex relative permittivity of the nanoantenna and the surrounding biological tissue, respectively. Here, the subscript p stands for “particle” and the subscript m stands for “medium.” In addition, $L_{i}$ denotes the geometrical factor of the prolate metal spheroid in each of the three Cartesian axes.^12^ $L_{i}$ ranges between 0 and 1 and satisfies $\sum_{i=1}^{3}L_{i}=1$.^15^ The exact values of $L_{i}$ depend on the major axis L (length) and minor axis D (diameter) of the prolate spheroidal nanoantennas [Fig. 1(b)]; the dependence can be visualized in Ref. 15. Both $\epsilon_{p}$ and $\epsilon_{m}$ are functions of the frequency of the RF irradiation, thereby leading to the frequency dependence of $F_{abs}$. The analytical expression of $F_{abs}$ is derived under the electrostatic approximation, which requires particles to be sufficiently small compared to the incident wavelength (see Sec. IV).

We begin our analysis of wireless RFA by plotting the frequency dependence of the real and imaginary parts of $\epsilon_{p}$ and $\epsilon_{m}$. Here, we confine our choices of the nanoantennas to gold nanowires for their appropriate approximation by a prolate spheroid, ease of synthesis and surface modification, favorable biocompatibility, and demonstrated photothermal applications.^16,17^ We apply the Drude model for free electrons (see Sec. IV) to calculate the negative real component and the imaginary component of the dielectric functions of two gold structures,^12^ as shown in Figs. 1(c) and 1(d), respectively. Specifically, the dielectric functions of bulk gold are determined by the intrinsic plasmon frequency and scattering rate with minimal surface effects. In contrast, gold nanowires with a diameter of 75 nm and a length of 25 *μ*m exhibit a lower magnitude for both permittivity and conductivity due to a smaller electron mean free path and thus a higher scattering rate in nanostructures (see Sec. IV).^15^ These gold nanowires can be readily synthesized with a seeded growth approach.^18^ When the aspect ratio of gold nanowires is not extremely large, the relative absorption ratio $F_{abs}$ is inversely dependent on $\epsilon_{p}$, thereby giving rise to a greater level of differential heating of the gold nanowires for the wireless RFA applications. We acknowledge that high-aspect-ratio nanowires synthesized in a laboratory may take geometries closer to cylinders than prolate spheroids, but we maintain the expressions for prolate spheroids even at high aspect ratios to increase the tractability of our calculations. Our future work will include calculations using polarizability expressions for cylinders. Additionally, while we consider a broad parameter space in length L and diameter D, extremely long and thin nanowires (e.g., L=100 *μ*m and D=10 nm) may be somewhat flexible, leading to bending *in vivo*. Such a curved geometry is beyond the scope of the theory considered in this manuscript, but it is important to consider that any deformation may affect the heating observed *in vivo*.

The dielectric functions of breast cancer tissue and the myocardium are shown in Figs. 1(e) and 1(f) based on interpolation of previous reports (see Sec. IV) and Gabriel parameterization, respectively.^19–26^ The breast cancer tissue represents an emerging target for RFA-assisted tumor ablation.^27^ Furthermore, the myocardium is the tissue target for RFA therapy to terminate cardiac arrhythmias.^28^ The dielectric functions of breast cancer tissue and the myocardium show similar values across the frequency range of interest, featuring a trend of decreasing magnitudes with increasing frequencies. While we acknowledge that the dielectric characteristics of biological tissues are inhomogeneous at the microscale,^29^ dielectric measurements for tissues are typically conducted for bulk tissue. Thus, we use dielectric measurements for bulk tissues throughout this manuscript.

### B. Wireless RFA in the breast cancer tissue

**FIG. 2.**
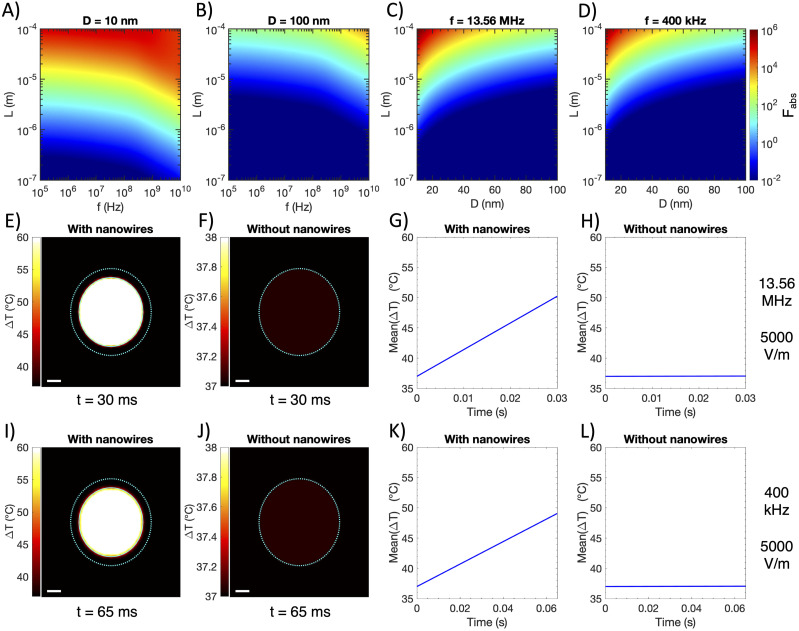
Wireless RFA in breast cancer tissue. [(a) and (b)] Relative absorption ratio $F_{abs}$ for gold prolate spheroids in breast cancer as a function of spheroid diameter D (marked in title), spheroid length L, and applied frequency f. (c) Relative absorption ratio $F_{abs}$ for gold prolate spheroids in breast cancer at f= 13.56 MHz as a function of spheroid diameter D and length L. (d) Relative absorption ratio $F_{abs}$ for gold prolate spheroids in breast cancer at f= 400 kHz as a function of spheroid diameter D and length L. (e)–(h) Simulations with 13.56 MHz field applied at 5000 V/m, showing temperature increase after 30 ms of heating breast tumor both with (e) and without (f) optimal nanowire bolus injection (tumor boundary marked with cyan dotted line, injection boundary marked with a cyan solid line), and average temperature of the nanowire-injected (g) and non-injected tumor (h) vs time. (i)–(l) Simulations with 400 kHz field applied at 5000 V/m, showing temperature increase after 65 ms of heating breast tumor both with (i) and without (j) optimal nanowire bolus injection (tumor boundary marked with a cyan dotted line, and injection boundary marked with a cyan solid line), and average temperature of the nanowire-injected (k) and non-injected tumor (l) vs time. Scale bar = 1 mm for all heatmaps.

With all parameters needed to calculate the relative absorption ratio $F_{abs}$, we ask if gold nanowires with a high aspect ratio can act as effective “radiothermal” agents to produce local, differential heating and RFA in the breast cancer tissue. Our recent findings suggest that prolate metal spheroids with a smaller diameter D and a greater length L result in a higher $F_{abs}$.^12^ We begin by varying the length L in a range from 100 nm to 100 *μ*m while setting the diameter D at 10 nm. We add another variable of RF frequency, which is tuned between 100 kHz, a frequency similar to that of the conventional RFA therapy,^1^ and 10 GHz, a frequency that shows the potential for producing differential heating in our recent work.^12^ The heatmap of $F_{abs}$ [Fig. 2(a)] reveals that differential heating occurs (i.e., $F_{abs}>1$) for 10-nm gold nanowires with a length of above ∼1 *μ*m. Moreover, these nanowires undergo significant differential heating when their lengths approach 100 *μ*m, with $F_{abs}$ being over 10^6^. These results suggest that extremely long gold nanowires can act as nanoantennas to concentrate the incident RF energy over 10^6^ times the absorption of the surrounding breast cancer tissue. In addition, the frequency dependence of $F_{abs}$ reveals that $F_{abs}$ is maximized around 13.56 MHz, a frequency commonly used in hyperthermia treatments.^11^ This higher-frequency irradiation is more effective in heating the same nanoantennas than the frequencies in the sub-MHz range, commonly applied in conventional RFA therapies.

To explore the optimum combination of the frequency, diameter, and length in a broad parameter space for wireless RFA, we calculate $F_{abs}$ for 100-nm diameter gold nanowires while varying the length L and RF frequency [Fig. 2(b)], and $F_{abs}$ of gold nanowires under fixed frequencies of 13.56 MHz and 400 kHz while varying their diameter D and length L [Figs. 2(c) and 2(d)]. We find much smaller $F_{abs}$ for 100-nm diameter gold nanowires across all frequencies and lengths, highlighting the importance of using narrow nanowires to concentrate RF energy most efficiently. Additionally, Figs. 2(c) and 2(d) confirm that longer and narrower gold nanowires absorb more RF energy and heat more than their shorter and wider counterparts, with 13.56 MHz irradiation representing a better frequency option for wireless RFA. In summary, the greatest differential heating in our parameter space occurs around 13.56 MHz for gold nanowires with D=10 nm and L=100 *μ*m in the breast cancer tissue.

With the optimized parameters of gold nanowires and the RF frequency, we next seek to find the temperature increases achievable with wireless RFA in the breast cancer tissue and compare those with the conventional RFA therapy and photothermal therapy to treat breast cancer. In our simulation, we consider a murine xenograft tumor model with a tumor volume of 100 mm^3^. We approximate the tumor’s geometry as a sphere. Gold nanowires with optimized parameters (D=10 nm, L=100 *μ*m, and $F_{abs}=1.06\times 10^{6}$ in the breast cancer tissue) are suspended in aqueous saline of 50 *μ*l spherical volume and injected intratumorally. The tumor size and injection volume are determined based on previous reports of photothermal tumor ablation.^30,31^ An experimentally achievable concentration of 10 mg/ml is used for gold nanowires in the simulation.^32^ After the intratumoral injection, gold nanowires are distributed within a spherical bolus of a 2.3 mm radius [marked with a cyan solid line in Figs. 2(e) and 2(i)], located at the center of the spherical tumor with a radius of 2.9 mm [marked with a cyan dotted line in Figs. 2(e), 2(f), 2(i), and 2(j)].

To solve the temperature increase in the breast cancer tissue induced by intratumorally injected gold nanowires, we solve the Pennes bioheat equation with a 13.56-MHz field and a 400-kHz field, both applied at 5000 V/m [Figs. 2(e)–2(l); see Sec. IV for details about calculation]. The recent device demonstrating spatially programmable RFA required voltages from 10 to 40 V at 400 kHz across electrodes with separation ∼2 mm.^3^ To enable comparison, we select the minimum field strength applied in Ref. 3, which we approximate as 5000 V/m (i.e., 10 V over 2 mm). We conduct our simulations at two RF frequencies, 13.56 MHz and 400 kHz. The frequency of 13.56 MHz demonstrates the greatest differential heating for 10-nm diameter and 100-*µ*m length gold nanowires in the breast cancer tissue. In addition, 400 kHz is a common frequency for clinical RFA and was applied for RFA in multifunctional balloon catheters.^3^ We begin our simulations with a uniform temperature distribution of 37 °C, and we apply RF fields until the center of the target region reaches ∼60 °C, at which point irreversible damage occurs in the tissue.^3^ A 2D cross-sectional view of the temperature distribution within the tumor reveals that 60 °C is reached in merely 30 ms [Fig. 2(e)], while neighboring tissues [and the non-injected tumor, Figs. 2(f) and 2(h)] heat by less than 0.1 °C due to nonspecific heating of the tissue by 13.56-MHz RF irradiation during this time. Time-dependent temperature dynamics indicates a rapid mean temperature increase of over 440 K/s [Fig. 2(g)], significantly faster than the maximum heating rate of ∼12 K/s reported in Fig. 3(a) of Ref. 3 and ∼0.2 K/s reported in Fig. 4(e) of Ref. 30. In addition, simulations performed at the 400-kHz frequency reveal that the 60 °C threshold is reached in only ∼65 ms [Fig. 2(i)], while surrounding tissues and the non-injected tumor heat by less than 0.1 °C over this period [Figs. 2(j) and 2(l)]. Furthermore, 400-kHz RF irradiation induces an initial mean temperature increase of ∼185 K/s [Fig. 2(k)], slightly lower than that induced by 13.56-MHz irradiation.

### C. Wireless RFA in the myocardium

**FIG. 3.**
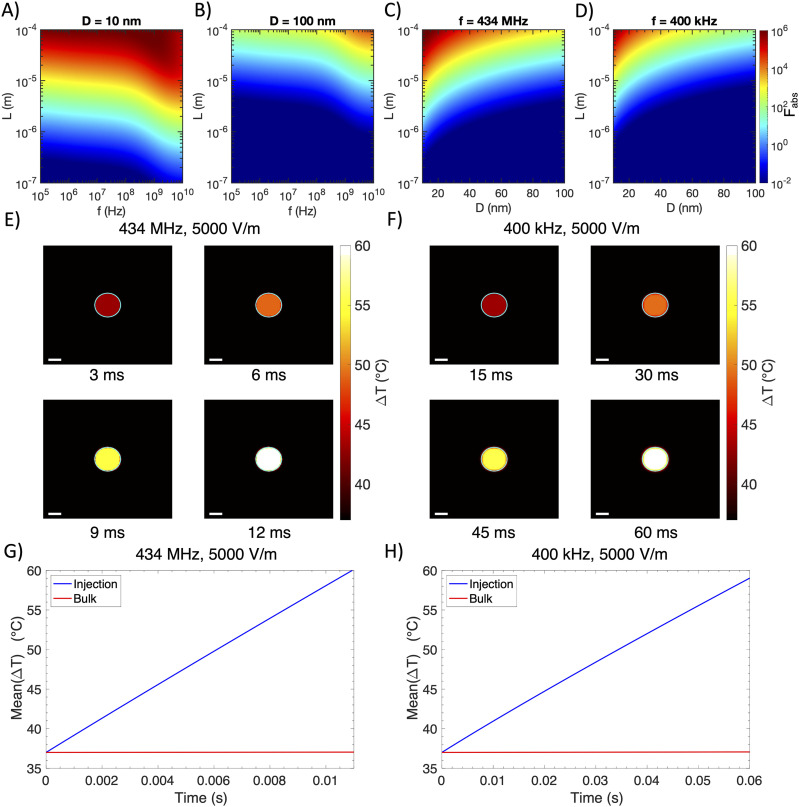
Wireless RFA in the myocardium. [(a) and (b)] Relative absorption ratio $F_{abs}$ for gold prolate spheroids in the myocardium as a function of spheroid diameter D (marked in title), spheroid length L, and applied frequency f. (c) Relative absorption ratio $F_{abs}$ for gold prolate spheroids in the myocardium at f= 434 MHz as a function of spheroid diameter D and length L. (d) Relative absorption ratio $F_{abs}$ for gold prolate spheroids in the myocardium at f= 400 kHz as a function of spheroid diameter D and length L. (e) Simulations with the 13.56 MHz field applied at 5000 V/m, showing temperature distribution over 12 ms of heating the myocardium with an optimal nanowire bolus injection (injection boundary marked with a cyan solid line), and (g) average temperature of the nanowire-injected and bulk tissue regions vs time. (f) Simulations with the 400 kHz field applied at 5000 V/m, showing temperature distribution over 60 ms of heating the myocardium with an optimal nanowire bolus injection (injection boundary marked with a cyan solid line), and (h) average temperature of the nanowire-injected and bulk tissue regions vs time. Scale bar = 1 mm for all heatmaps.

We next ask if gold nanowires can absorb free-space RF irradiation to enable wireless RFA in the myocardium for treating cardiac arrhythmias. RFA therapy is the most widely used procedure to terminate cardiac arrhythmias by creating lesions to destroy abnormal electrical pathways.^33^ We first calculate $F_{abs}$ for gold nanowires with a fixed diameter of 10 nm by varying their length L and the incident RF frequency [Fig. 3(a)]. Similarly to the calculation of $F_{abs}$ in the breast cancer tissue, we find that an increased length L and a higher frequency produce greater differential heating over 10^6^. In particular, the heating is maximized around 434 MHz, another common frequency used in hyperthermia treatments.^11^ In contrast, 100-nm diameter gold nanowires only produce differential heating up to $1.5\times 10^{4}$ in the same parameter space [Fig. 3(b)]. Fixing the frequency at 434 MHz and 400 kHz, longer and narrower nanowires act as more efficient nanoantennas to absorb RF energy, with those of a 10-nm diameter and a 100-*µ*m length in 434 MHz fields producing the greatest differential heating in the myocardium [Figs. 3(c) and 3(d)].

It is instructive to calculate the absorption efficiency of an optimized nanoantenna, defined here as the ratio of a nanowire’s absorption cross section to its cross-sectional area,^15^

$$Q_{abs}=\frac{4C_{abs}}{\pi LD}.$$ (2)

Here, the cross-sectional area is given by πLD/4 because absorption is maximized when a nanowire’s long axis is aligned with the electric field vector, and thus, its long axis is perpendicular to the field’s propagation vector. For a nanowire in the myocardium at 434 MHz with L=100 *μ*m and D=10 nm, we calculate $Q_{abs}=1.2$. This value of $Q_{abs}>1$ means that a nanoantenna presents a larger target to the incoming RF than its cross-sectional area alone. In fact, field lines of the Poynting vector that would otherwise have passed nearby the nanowire unimpeded are “deflected” toward the surface of the nanowire.^15^ Reference 15 (pp. 340–341) contains a more thorough description of this phenomenon.

The optimized parameters of gold nanowires allow us to quantitatively evaluate the spatial distribution and temporal dynamics of heating in the myocardium and assess the feasibility of this approach for treating myocardial arrhythmias. We use a Langendorff rabbit heart model as described previously^3^ in our simulations. Specifically, gold nanowires are spatially distributed within a diameter of 2 mm, similar to the distance between a pair of electrodes for RFA in the multifunctional balloon catheter.^3^ Furthermore, we use the same concentration of gold nanowires as used in breast cancer simulations above. We conduct simulations under two RF frequencies, 434 MHz and 400 kHz, each at 5000 V/m. With incident RF waves of 434-MHz frequency, spatiotemporal temperature mapping for a 10 × 10 mm^2^ region surrounding the gold nanowires shows efficient heating within the region of injected nanoantennas [Fig. 3(e)], with temperature increases nearly three orders of magnitude faster than that shown in Fig. 5(f) of the recent multifunctional balloon catheter paper.^3^ In addition, spatiotemporal mapping of the temperature increase under 400-kHz irradiation reveals similarly rapid heating and high spatial resolution, although not as rapid as 434 MHz [Fig. 3(f)]. We then plot the average temperature within the 1 mm injection radius as a function of time and observe rapid temperature increases of ∼2100 and ∼360 K/s under 434-MHz and 400-kHz irradiation, respectively, which are significantly higher than those of ∼3 and ∼1 K/s for the bulk myocardial tissue [Figs. 3(g) and 3(h)]. We find that our simulated temperature increase rate during wireless RFA is significantly faster than that measured with the multifunctional balloon catheter, enabling shorter RF application durations and consequently reducing heat diffusion to surrounding healthy tissues.

## III. DISCUSSION AND CONCLUSIONS

Our simulations confirm the feasibility of using free-space RF irradiation to produce efficient, localized thermal ablation in biological tissues with high spatial resolution. The efficiency and spatial localization of wireless RFA are facilitated by gold nanowires with optimized geometries, which act as “radiothermal” nanoantennas to strongly absorb incident RF energy and produce significant differential heating over the surrounding tissue. We study the feasibility of wireless RFA in two tissue targets, breast cancer tissue and the myocardium, which are commonly targeted by conventional RFA for treating breast cancer and myocardial arrhythmias, respectively. Several frequencies of the electromagnetic radiation are chosen in the study: 13.56 and 434 MHz represent the greatest heating efficiency in our parameter spaces of interest, while 400 kHz represents an identical frequency as conventional RFA therapy. Owing to the free-space propagation and deep-tissue penetration of RF irradiation,^11^ wireless RFA offers a much less invasive, non-contact alternative to conventional RFA approaches.

Wireless RFA relies on gold nanowires as nanoantennas to concentrate the incident RF irradiation and produce local heating. Compared to conventional RFA electrodes, the longest dimension (100 *μ*m) of wireless RFA nanoantennas is 5x smaller than the length of exposed electrodes, while their shortest dimension (10 nm) is nearly five orders of magnitude smaller than the width of RFA electrodes. The much smaller footprint of RFA nanoantennas enables their injectability through a standard medical syringe outfitted with a hypodermic needle, thus imposing minimal invasiveness to the tissue target. Although we have confined our simulations to intratumorally or epicardially injected nanoantennas for achieving a high local concentration, intravenous injection may offer an even less invasive route for *in vivo* administration. Intravenously delivered nanoparticles have shown great promise as imaging and neuromodulation agents.^34–36^ Furthermore, intravenously delivered gold nanoshells showed promising oncological and functional outcomes in a clinical trial of nanoparticle-based photothermal cancer therapy. Specifically, intravenously injected gold nanoshells accumulate in the prostate tumors as a result of increased vascular fenestrations and permeability, aberrant neovasculature, and decreased lymphatic drainage in the tumor.^37^ Despite some recent studies challenging the enhanced permeability and retention effect for delivering anticancer nanoparticles to solid tumors,^38,39^ coating radiothermal nanoantennas with tumor-penetrating peptides may provide an alternative approach for achieving tumor accumulation of these nanoantennas after intravenous injection.^40^ Compared to the clinical trial of photothermal cancer therapy with gold nanoshells, our wireless RFA approach may achieve efficacious ablation of the cancerous tissue deep inside the human body without the invasive insertion of optical fibers.^37^ Compared to conventional RFA therapy, our wireless RFA approach can ablate cancerous and dysfunctional tissues with much reduced invasiveness to the overlying and surrounding healthy tissues for treating a wide array of diseases ranging from cancers, cardiac arrhythmias, to hypertension.

## IV. METHODS

### A. Relative absorption ratio calculations

We use the parameters $\omega_{p}$ and $\gamma_{bulk}$ reported in Ref. 41 for gold to calculate $\epsilon_{p}$ for gold prolate spheroids using the Drude model for free electrons. For convenience, these values are reproduced in Table I. The Drude model for free electrons is given by

$$\epsilon_{p}=1-\frac{{\omega_{p}}^{2}}{\omega \left(\omega +i\gamma \right)}.$$ (3)

We apply scattering rate corrections due to the finite size of all nanoparticles considered in this manuscript. In particular, we correct the scattering rate using the formula^15^

$$\gamma =\gamma_{bulk}+\frac{v_{F}}{L_{eff}},$$ (4)

where $v_{F}$ is the Fermi velocity within a metal (reproduced in Table I) and $L_{eff}$ is the effective mean free path within the nanoparticle. This correction term accounts for electron scattering off the surface of a nanoparticle. We rely on a detailed analysis employing geometrical probability to calculate $L_{eff}$.^42^ For prolate spheroids with length L and diameter D, the effective mean free path $L_{eff}$ is given by^42^

$$L_{eff}=\frac{2D}{3/2+3F_{2}/2},\;F_{2}=\frac{sin^{-1}\left(e\right)}{e},\;e=\sqrt{1-{\left(D/L\right)}^{2}}.$$ (5)

**TABLE I.** Input parameters for the Drude model for Au.

Parameter	Value	Reference
$\omega_{p}$ (rad/s)	$1.37\times 10^{16}$	41
$\gamma_{bulk}$ (rad/s)	$4.05\times 10^{13}$	41
$v_{F}$ (m/s)	$1.39\times 10^{6}$	48

Corrections to the scattering rate can have a significant impact on the dielectric function of small nanoparticles. As an example, we consider a 10 nm diameter spherical gold nanoparticle. The expressions in Eq. (5) reduce to $L_{eff}=\frac{2D}{3}$ for spherical particles, and we subsequently calculate an ∼38 times smaller ${\epsilon_{p}}^{\prime }$ and ∼6 times smaller ${\epsilon_{p}}^{\prime \prime }$ for the nanoparticle compared to bulk gold (using the parameters in Table I).

While a Gabriel parameterization for the myocardium exists (for calculating $\epsilon_{m}$), such a parameterization does not exist for breast cancer. To calculate the complex dielectric function $\epsilon_{m}$ for breast cancer tissues across the spectrum from 100 kHz to 10 GHz, we interpolate between the datasets of Surowiec^20^ and Cheng.^19^ In Fig. S1, we plot these datasets alongside the interpolated curve we used to calculate $\epsilon_{m}$.

### B. Validity of the electrostatic approximation

The analytical expression of $F_{abs}$ in Eq. (1) is derived in Ref. 12 under the electrostatic approximation. This approximation is only valid for nanoparticles that meet certain conditions (discussed in detail in Ref. 15). Namely, if a spheroidal nanoparticle’s longest axis (with length a) is aligned with the direction of a propagating wave, the particle and medium must satisfy the following conditions:^15^

$$\frac{\omega }{c_{0}}{n_{m}}^{\prime }a\ll 1\;and\;\frac{\omega }{c_{0}}{n_{m}}^{\prime \prime }a\ll 1,$$ (6)

$$\frac{\omega }{c_{0}}{n_{p}}^{\prime }a\ll 1\;and\;\frac{\omega }{c_{0}}{n_{p}}^{\prime \prime }a\ll 1,$$ (7)

where the complex refractive indices for tissue and nanoparticles are defined as $n_{m}=\sqrt{\epsilon_{m}}={n_{m}}^{\prime }+i{n_{m}}^{\prime \prime }$ and $n_{p}=\sqrt{\epsilon_{p}}={n_{p}}^{\prime }+i{n_{p}}^{\prime \prime }$, respectively. Additionally, ω represents the angular frequency of the incident wave, and $c_{0}$ represents the speed of light in free space. In the plots in this manuscript, we set 0.1 to be the maximum value that satisfies ≪1.^12^ If a nanoparticle violates the electrostatic approximation with one of its axes, we set the term in the sum in Eq. (1) corresponding to that axis equal to zero. By doing so, the calculated values of $F_{abs}$ in this manuscript represent a lower bound on the relative absorption ratio. Beneficially, the analytical expressions enabled by the electrostatic approximation are straightforward to evaluate for users in laboratories or clinics.

### C. Thermal simulations

We use the bioheatExact function in the k-Wave toolbox (MATLAB software) to solve the Pennes bioheat equation in three dimensions.^43^ The Pennes bioheat equation is given by

$$\rho C\frac{dT}{dt}=k\nabla^{2}T+\rho_{b}W_{b}C_{b}\left(T-T_{b}\right)+Q,$$ (8)

where ρ is the tissue density, C is the tissue specific heat capacity, T is the tissue temperature, k is the tissue thermal conductivity, $\rho_{b}$ is the blood density, $W_{b}$ is the blood perfusion rate, $C_{b}$ is the blood specific heat capacity, $T_{b}$ is the arterial blood temperature, and Q is the power density contributed by radio frequency absorption. Q is calculated separately for injected and non-injected regions. For non-injected regions, $Q=\frac{1}{2}\sigma E^{2}$, where σ is the conductivity of the background tissue (in S/m) and E is the applied electric field strength (in V/m). For injected regions, $Q=\frac{1}{2}\sigma E^{2}\times \left(5.18\times 10^{-4}F_{abs}\right)$, where $5.18\times 10^{-4}$ is the volume fraction of gold in a 10 mg/ml saline suspension and $F_{abs}$ is the relative absorption ratio of a single gold prolate spheroid compared to the background tissue.

We refer to Ref. 44 for the thermal properties of both breast cancer and healthy breast tissue. We refer to Refs. 45 and 46 for the thermal properties of myocardium. We refer to Ref. 47 for the thermal properties of the blood. For convenience, these values are reproduced in Table II. The domain size for all simulations is 10 × 10 × 10 mm^3^, and we use a mesh with 0.1 mm step size to discretize the domain.

**TABLE II.** Parameters used in Pennes bioheat calculations.

Parameter	Value	Reference
ρ (healthy breast tissue)	1080 kg/m^3^	44
C (healthy breast tissue)	3000 J/(kg K)	44
k (healthy breast tissue)	0.48 W/(m K)	44
$W_{b}$ (healthy breast tissue)	0.00018 s^−1^	44
ρ (breast cancer)	1080 kg/m^3^	44
C (breast cancer)	3500 J/(kg K)	44
k (breast cancer)	0.48 W/(m K)	44
$W_{b}$ (breast cancer)	0.009 s^−1^	44
ρ (myocardium)	1086 kg/m^3^	45
C (myocardium)	3669 J/(kg K)	45
k (myocardium)	0.55 W/(m K)	45
$W_{b}$ (myocardium)	0.017 s^−1^	46
$\rho_{b}$ (all tissues)	1050 kg/m^3^	47
$C_{b}$ (all tissues)	3617 J/(kg K)	47
$T_{b}$ (all tissues)	37.15 K	47

The bioheatExact function assumes periodic boundary conditions. Although these boundary conditions prevent heat from leaving the domain, this is a good assumption over the ∼100 ms timescales used in the simulations. Using the thermal diffusivity of water (D= 1.4 × 10^−7^ m^2^/s, water used to approximate tissue), the characteristic diffusion length $L_{D}$ over 100 ms is given by $L_{D}=\sqrt{4Dt}\approx 0.2$ mm. Thus, the intense heat generated within the injection region in the center of the domain will not have time to reach the boundaries of the simulation over the timescales considered here.

## SUPPLEMENTARY MATERIAL

The supplementary material section includes Fig. S1 representing interpolated curves based on the data of Surowiec^20^ and Cheng^19^ used to calculate the complex dielectric function for breast cancer across the frequency spectrum from 100 kHz to 10 GHz.

## Data Availability

The data that support the findings of this study are available from the corresponding author upon reasonable request.
